# Acquired resistance to decitabine and cross-resistance to gemcitabine during the long-term treatment of human HCT116 colorectal cancer cells with decitabine

**DOI:** 10.3892/ol.2015.3253

**Published:** 2015-05-22

**Authors:** MIKA HOSOKAWA, MAI SAITO, AIKO NAKANO, SAKURA IWASHITA, AYANO ISHIZAKA, KUMIKO UEDA, SEIGO IWAKAWA

**Affiliations:** Department of Pharmaceutics, Kobe Pharmaceutical University, Higashinada-ku, Kobe 658-8558, Japan

**Keywords:** colorectal cancer, decitabine, epigenetics, gemcitabine, resistance

## Abstract

The aim of the present study was to determine the effects of long-term exposure of decitabine (DAC) to HCT116 colorectal cancer (CRC) cells on the acquisition of resistance to DAC as well as cross-resistance to anticancer drugs used for CRC or other epigenetic modifiers. In the present study, DAC-resistant HCT116 CRC cells were established through long-term treatment with increasing concentrations of DAC (10 to 540 nM); and the cross-resistance to other drugs was subsequently examined. DAC-resistant HCT116 cells were obtained following a 104-day treatment with DAC, including DAC-free intervals. The results demonstrated that the IC_50_ value of DAC was increased ~100-fold in DAC-resistant HCT116 cells. Messenger (m)RNA expression of *secreted frizzed-related protein 1 (SFRP1)*, which is regulated by DNA methylation, was not detected in DAC-resistant cells; however, *SFRP1* mRNA was present in HCT116 cells treated with DAC for 52 days. DNA methyltransferase 1 (DNMT1) protein levels were slightly decreased until day 81 and then returned to control levels in DAC-resistant cells. Further experiments using DAC-resistant HCT116 cells revealed that these cells exhibited cross-resistance to gemcitabine (Gem); however, cross-resistance was not observed for other DNMT inhibitors (azacitidine and zebularine), histone deacetylase inhibitors (trichostatin A, vorinostat and valproic acid) or anticancer drugs for CRC (5-fluorouracil, irinotecan and oxaliplatin). Furthermore, the protein expression levels of cytidine deaminase (CDA) were increased, while those of deoxycytidine kinase (dCK) were decreased in DAC-resistant HCT116 cells; by contrast, the mRNA expression levels for these proteins were not significantly altered. In conclusion, the results of the present study indicated that the long-term treatment of HCT116 cells with DAC led to the acquisition of resistance to both DAC and Gem. In addition, these results may be partly attributed to changes in CDA and/or dCK, which are involved in metabolic pathways common to these two drugs.

## Introduction

Cancer is known to be caused by genetic as well as epigenetic disorders. Cancers often exhibit aberrant methylation of gene promoter regions in key tumor-suppressor genes, which consequently results in the loss of gene functions (1). DNA hypomethylating agents, including decitabine (DAC) and azacitidine (AC), have been approved for the treatment of myelodysplastic syndromes (MDS) (2). These two drugs are analogs of cytidine, which trap the DNA methyltransferase 1 (DNMT1) protein via covalent interactions (3). DNMT1 depletion results in passive demethylation in dividing cells as well as the re-expression of critical genes, which were silenced through aberrant promoter hypermethylation. Prolonged re-expression of these genes has been reported to be associated with clinical responses (1). DNMT1 inhibitors, including DAC and AC, have been demonstrated to disrupt DNA methylation through inhibiting DNMT1 at low concentrations; however, these inhibitors were reported to induce cytotoxicity at high concentrations (3,4).

Resistance to anticancer drugs is a critical problem that limits the effectiveness of chemotherapy (5). Resistance generally develops with long-term exposure. Cancer cells that acquire resistance to one anticancer drug may also become simultaneously resistant to different drugs; this has been referred to as multidrug-resistance or cross-resistance (6). Other epigenetic modifiers, such as histone deacetylase (HDAC) inhibitors, which include trichostatin A (TSA), vorinostat (suberoylanilide hydroxamic acid; SAHA) and valproic acid (VPA), have been reported to induce moderate resistance and cross-resistance to different HDAC inhibitors in colon cancer cells (7–9). By contrast, these colon cancer cells that acquired resistance to HDAC inhibitors retained their sensitivity to non-HDAC inhibitor-type anticancer drugs. However, it remains to be elucidated whether DNMT inhibitors exhibit cross-resistance. It was reported that a high cytidine deaminase (CDA) to deoxycytidine kinase (dCK) ratio may be a marker of primary resistance to DAC in MDS (10). Previous studies have demonstrated that resistance to DAC in HL60 cells was induced by exposing these cells to DAC for 14 days and was associated with attenuated dCK levels due to dCK mutations; however, dCK mutations were not detected in MDS patients following relapse (10,11). A concentration of 10 µM DAC was used in a previous study (11), which was markedly higher than that used to inhibit DNMT1 (~0.2 µM). However, it remains to be elucidated whether long-term treatment with DAC, at concentrations that inhibit DNMT1, results in cell resistance. A previous study demonstrated that resistance to AC was acquired by perturbing its activation with uridine-cytidine kinase (UCK) 2 gene mutations following treatment with increasing concentrations of AC (0.2–1.0 µM) (12). CDA, which inactivates DNMT inhibitors, may also contribute to poorer outcomes with AC or DAC therapy (13). Previous studies have reported that human nucleoside transporters, including equilibrative nucleoside transporters (ENTs) and concentrative nucleoside transporters (CNTs), had an important role in the uptake and cytotoxicity of DAC and AC (14–16). However, the underlying mechanisms for resistance to DAC are poorly understood in terms of transporters as well as enzymes. Furthermore, information on the treatment of solid tumors, such as colorectal cancer (CRC), with DAC is limited and at present, has only resulted in a limited number of responses (17). By contrast, combination therapy with DAC and traditional cytotoxic anticancer drugs may be beneficial for solid tumors (18,19). However, cross-resistance in DAC-resistant cancer cells has not yet been examined in detail.

In the present study, DAC resistance was established in HCT116 CRC cells through long-term treatment with increasing concentrations of DAC. It was previously demonstrated that HCT116 cells were the most sensitive to DAC among four human CRC cell lines (20). HCT116 cells, classified as cytosine-phosphate-guanine island methylator phenotype (CIMP)-positive, are an extensively studied line of human CRC cells in the field of epigenetics (21,22). Cancers with CIMP exhibit aberrant DNA methylation, leading to the concordant promoter hypermethylation of multiple genes (23). The present study aimed to determine the effects of long-term exposure of HCT116 cells to DAC, at concentrations that inhibit DNMT1, on the acquisition of resistance to DAC, in terms of cytotoxicity and gene expression regulated by DNA methylation. In addition, the present study investigated whether DAC-resistant HCT116 cells exhibited cross-resistance to anticancer drugs used for CRC or other epigenetic modifiers.

## Materials and methods

### 

#### Materials

The human HCT116 colon carcinoma cell line was purchased from DS Pharma Biomedical Co., Ltd (Osaka, Japan). McCoy's 5A medium, Leibovitz L-15 medium (L-15), penicillin-streptomycin and fetal bovine serum (FBS) were purchased from Life Technologies (Carlsbad, CA, USA). Oxaliplatin (L-OHP), gemcitabine (Gem), DAC, AC, zebularine (Zeb), TSA and SAHA were purchased from Wako Pure Chemical Industries, Ltd (Osaka, Japan). 5-fluorouracil (5-FU) was purchased from Nacalai Tesque, Inc. (Kyoto, Japan). Irinotecan (CPT-11), 7-ethyl-10-hydroxycamptothecin (SN-38) and VPA sodium salt were purchased from Sigma-Aldrich (St. Louis, MO, USA). Monoclonal mouse anti-β-actin (dilution, 1:1,000; catalog no. sc-4778), monoclonal mouse anti-CDA (dilution, 1:1,000; catalog no. sc-365292), goat anti-mouse immunoglobulin (Ig)G-horseradish peroxidase (HRP; dilution, 1:2,000–5,000; catalog no. sc-2005) and goat anti-rabbit IgG-HRP antibodies (dilution, 1:2,000; catalog no. sc-2004) were purchased from Santa Cruz Biotechnology, Inc. (Dallas, TX, USA). Monoclonal mouse anti-DNMT1 (dilution, 1:1,000; catalog no. ab-13537), polyclonal rabbit anti-ENT1 (dilution, 1:1,000; catalog no. ab-48607) and polyclonal rabbit anti-dCK (dilution, 1:500; catalog no. ab-91599) antibodies were purchased from Abcam (Cambridge, UK). All other chemicals were of the highest grade commercially available.

#### Cell culture and establishment of DAC-resistant HCT116 cells

HCT116 cells were grown in McCoy's 5A medium supplemented with 10% FBS, 100 U/ml penicillin and 100 µg/ml streptomycin at 37°C in 5% CO_2_-95% air. DAC-resistant HCT116 cells were generated through long-term treatment with increasing concentrations of DAC (10–540 nM) including DAC-free intervals in order to allow the surviving cells to recover. HCT116 cells were initially seeded at a density of 3×10^3^ cells/100-mm dish and subcultured every 14 days with DAC treatment from day 7 to day 12. This protocol was repeated twice (DAC concentration, cycle 1, 10 nM; cycle 2, 30 nM). After 28 days, the cells were seeded at a density of 6×10^5^ cells/100-mm dish and treated with DAC after 24 h. After another 72 h, the cells were subcultured and cultured in DAC-free medium for 96 h to allow the surviving cells to recover. This protocol was repeated 9 times until day 104. The additional DAC-free intervals after DAC treatment for 72 h were 24 h for cycle 7 and 9, and 72 h for cycle 8. The concentrations of DAC were 60 nM for cycle 1–3, 240 nM for cycle 4–7, 360 nM for cycle 8 or 540 nM for cycle 9.

#### Cytotoxicity assay

A cytotoxicity assay was performed using the water-soluble tetrazolium salt-8 (WST8) assay with a Cell Counting kit-8 (Dojindo Laboratories, Kumamoto, Japan). Cells (2×10^3^/well) were seeded onto a 96-well plate in 100 µl McCoy's 5A culture medium supplemented with 10% FBS, 100 U/ml penicillin, and 100 µg/ml streptomycin at 37°C in 5% CO_2_-95% air. After 24 h, drugs diluted with the culture medium were added to each well. The concentrations of the drugs were as follows: DAC (0.1, 0.4, 1.6, 6.3 or 25.0 µM for control, day 28 or day 52; 25, 100, 250, 500, 1000 or 2000 µM for day 81, day 104); AC (3.1, 6.3, 12.5, 25.0, 50.0 or 100.0 µM); Zeb (63, 125, 250, 500, 100, 1333 or 2000 µM); TSA (6.3, 12.5, 16.7, 25.0 or 50.0 nM); SAHA (0.3, 0.4, 0.6, 0.8, 1.3, 2.5, 5.0 or 10.0 µM); VPA (0.6, 0.8, 1.3, 1.7, 2.5 or 5.0 mM); 5-FU (0.1, 0.4, 1.6, 6.3, 25.0 or 100.0 µM); CPT-11 (0.9, 1.9, 3.8, 7.5, 15.0 or 30.0 µM); SN-38 (0.6, 1.3, 2.5, 5.0, 10.0, 20.0 or 40.0 nM), L-OHP (0.3, 0.4, 0.6, 0.8, 1.3, 2.5, 5.0 or 10.0 µM) or Gem (1.6, 2.4, 3.6, 5.3, 8.0, 12.0 or 36.0 nM for control cells;12, 37, 111, 333, 1000, 3000 nM for DAC-resistant cells). Following drug treatment for 72 h, cells were washed with L-15 medium and incubated with 100 µl L-15 medium (due to the high background absorbance of McCoy's 5A medium) and 10 µl WST-8 solution for 1–2 h at 37°C. The conversion of WST-8 to formazan by living cells (active mitochondria) was measured at 450 nm for the indicator color and 655 nm for the background using a Bio-Rad 550 Microplate Reader (Bio-Rad Laboratories, Inc., Hercules, CA, USA). In all assays, reactions containing no cells were used to determine blank values, which were subtracted from values obtained from the assays with cells. The IC_50_ values of drugs in cells were calculated according to the sigmoid inhibitory effect model: *E*=(*E*_max_ xIC_50_^γ^)/(C^γ^+IC_50_^γ^), by means of a nonlinear least-squares fitting method (Solver, Microsoft Excel 2010; Microsoft Corp., Redmond, WA, USA). *E* and *E*_max_ represent the surviving fraction (% of control) and the maximum surviving fraction, respectively; *C* and γ represent the drug concentration in medium and sigmoid factor, respectively, as described previously (24).

#### Reverse transcription quantitative polymerase chain reaction (RT-qPCR)

Total RNA was extracted from cell lines using RNeasy Mini kits (Qiagen, Inc., Valencia, CA, USA) according to the manufacturer's instructions for mammalian cells. Total RNA was reverse-transcribed into complementary DNA using a ReverTra Ace qPCR RT Master Mix with gDNA Remover (Toyobo Co., Ltd., Osaka, Japan). qPCR was performed on a Rotor-Gene Q (Qiagen, Inc.) using SYBR Green (Toyobo Co., Ltd.). The PCR conditions were as follows: Initial denaturation for 1 cycle of 1 min at 95°C, followed by 40 cycles of 10 sec at 95°C (denaturation), 10 sec at 60°C (annealing) and 20 sec at 72°C (extension). Following these cycles, a melting curve was used to confirm the single product. The expression levels of each messenger (m)RNA were normalized to that of *ribosomal protein L27* (*RPL27*), as a housekeeping gene (25). Relative expression levels of the target genes were expressed as 2^−ΔCt^ (26). The primers used in the present study were obtained from Life Technologies and the sequences were as follows: Human *RPL27* forward, 5′-ATCGCCAAGAGATCAAAGATAA-3′ and reverse, 5′-TCTGAAGACATCCTTATTGACG-3′; human *SFRP1* forward, 5′-AATGCCACCGAAGCCTCCAAGC-3′ and reverse, 5′-TCATCCTCAGTGCAAACTCGCTG-3′; human *ENT1* forward, 5′-AGG AGC CAA GAG CAG GCA AAG AG-3′ and reverse, 5′-ACA GTC ACG GCT GGA AAC ATC CC-3′; human *CDA* forward, 5′-ACAGTCACTTTCCTGTGGGGGC-3′ and reverse, 5′-AGCGGTCCGTTCAGCACAGATG-3′; and human *dCK* forward, 5′-AAGCTGCCCGTCTTTCTCAGCC-3′ and reverse, 5′-TTCCCTGCAGCGATGTTCCCTTC-3′.

#### Western blot analysis

Nuclear (DNMT1, CDA and dCK) or whole-cell (ENT1) proteins were isolated using Mammalian Protein Extraction Reagent (Thermo Scientific, Rockford, IL, USA) or Radioimmunoprecipitation Assay Buffer (Nacalai Tesque, Inc.), respectively. Protein concentrations were measured using the Quant-iT Protein Assay kit (Molecular Probes, Life Technologies). Protein samples (20 µg) were separated by electrophoresis using 4–12% (DNMT1) or 10% (ENT1, CDA and dCK) NuPAGE Bis-Tris gel (Invitrogen Life Technologies, Carlsbad, CA, USA) with 3-propanesulfonic acid or 2-ethanesulfonic acid buffer (Invitrogen Life Technologies), respectively, and transferred to a polyvinylidene fluoride membrane using iBlot (Invitrogen Life Technologies). The membranes were blocked with Blocking One (Nacalai Tesque, Inc.) at room temperature for 30 min and incubated with primary antibodies (monoclonal mouse anti-β-actin, monoclonal mouse anti-DNMT1, polyclonal rabbit anti-ENT1, monoclonal mouse anti-CDA or polyclonal rabbit anti-dCK, as aforementioned) for 1 h at room temperature. The membranes were washed with Tris-buffered saline-0.1% Tween 20 and incubated with the secondary antibodies (goat anti-mouse IgG-HRP or goat anti-rabbit IgG-HRP, as aforementioned) for 1 h at room temperature. The proteins were visualized using Chemi-Lumi One Super (Nacalai Tesque, Inc.). Relative band intensities were estimated using Image J software, version 1.48 (National Institute of Health, Bethesda, MD, USA).

#### Statistical analysis

All values are expressed as the mean ± standard error of the mean. Differences between two groups were evaluated using the unpaired Student's *t*-test. One way analysis of variance followed by post-hoc analysis was used for data with >2 groups. P<0.05 was considered to indicate a statistically significant difference between values.

## Results

### 

#### Acquired DAC resistance following long-term treatment of HCT116 cells with DAC

**Figure 1.**
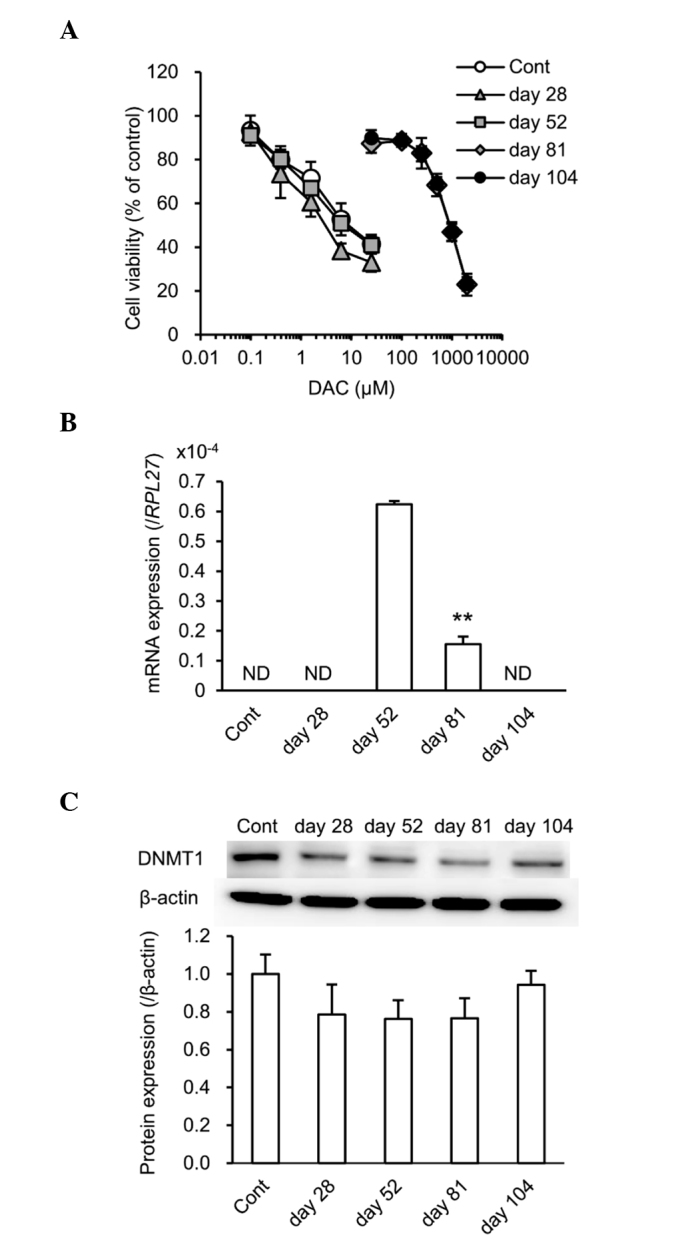
HCT116 cells acquire resistance to DAC following DAC treatment for 104 days. (A) HCT116 cells treated with DAC for 28, 52, 61 and 104 days were incubated with various concentrations of DAC for 72 h in a 96-well plate. Cell viability was measured using the water-soluble tetrazolium salt-8 assay. (B) *SFRP1* mRNA expression in control and DAC-treated HCT116 cells was analyzed by reverse transcription quantitative polymerase chain reaction and normalized to *RPL27* mRNA expression. **P<0.01 vs. day 52. (C) DNMT1 protein expression in control and DAC-treated HCT116 cells was analyzed by western blotting. β-actin was used as a loading control. Band intensities were then quantified using Image J software. Values are presented as the mean ± standard error of the mean of three independent experiments. DAC, decitabine; *SFRP1*, *secreted frizzled-related protein 1*; *RPL27*, *ribosomal protein 27*; ND, not detected; Cont, control; DNMT1, DNA methyltransferase 1.

HCT116 cells were treated with increasing concentrations of DAC for 104 days. The sensitivity of HCT116 cells to DAC was then examined (Fig. 1A; Table I). The cell viability of HCT116 cells treated with DAC for 81 or 104 days was markedly increased compared with the control cells. The growth curve of HCT116 cells treated with DAC for 81 or 104 days shifted to higher concentration of DAC compared with the control cells (Fig. 1A). As presented in Table I, IC_50_ values in HCT116 cells increased ~100-fold following treatment with DAC for >80 days compared with the control group, indicating that HCT116 cells became resistant to DAC. This resistance was stable over a period of 4 weeks even when the cells were cultured in the absence of DAC (data not shown). In addition, mRNA expression levels of *secreted frizzed-related protein 1* (*SFRP1*), which is known to be regulated by DNA methylation, were determined (21,27). As shown in Fig. 1B, mRNA expression of *SFRP1* was not detected in control HCT116 cells; however, its expression was present following treatment with DAC for 52 days, indicating that DAC exhibited a DNA demethylation effect by day 52. However, this effect was attenuated following subsequent treatment with DAC, as *SFRP1* mRNA expression levels were decreased significantly by day 81 (P<0.01) and were absent by day 104. The protein expression of DNMT1, the target of DAC, is shown in Fig. 1C (3). DNMT1 protein levels were decreased until day 81 and then returned to control levels by day 104; however, no significant differences were observed (P>0.05; Fig. 1C). HCT116 cells treated with DAC for 104 days were used as DAC-resistant HCT116 cells for all subsequent experiments.

#### Absence of cross-resistance to epigenetic modifiers in DAC-resistant HCT116 cells

In order to determine whether DAC-resistant HCT116 cells acquired cross-resistance to other epigenetic modifiers, their sensitivity to various epigenetic modifiers, including AC, Zeb, TSA, SAHA and VPA, was investigated. Although a significant difference was observed in the IC_50_ value of VPA (P<0.05), the IC_50_ values of the other epigenetic modifiers examined did not change markedly, indicating that none of epigenetic modifiers demonstrated cross-resistance to DAC (Table II).

#### Cross-resistance to anticancer drugs in DAC-resistant HCT116 cells

In order to investigate whether DAC-resistant HCT116 cells acquired cross-resistance to other anticancer drugs, their sensitivity to various anticancer drugs used to treat CRC, including 5-FU, CPT-11, SN-38, L-OHP and Gem, were determined. Although a significant difference was observed in the IC_50_ values of CPT-11 and SN-38 (P<0.05), the IC_50_ values of anticancer drugs for CRC, 5-FU, CPT-11, SN-38 and L-OHP, did not change markedly, indicating that cross-resistance was not exhibited for these drugs (Table III). By contrast, the IC_50_ value of Gem, the transport and metabolic pathways of which are similar to those of DAC (28), increased 32-fold. This result indicated that common factors between DAC and Gem, including ENT1, CDA and dCK, may be involved in the acquired resistance to DAC by HCT116 cells.

#### Alterations in CDA and dCK protein expression levels in DAC-resistant HCT116 cells

**Figure 2.**
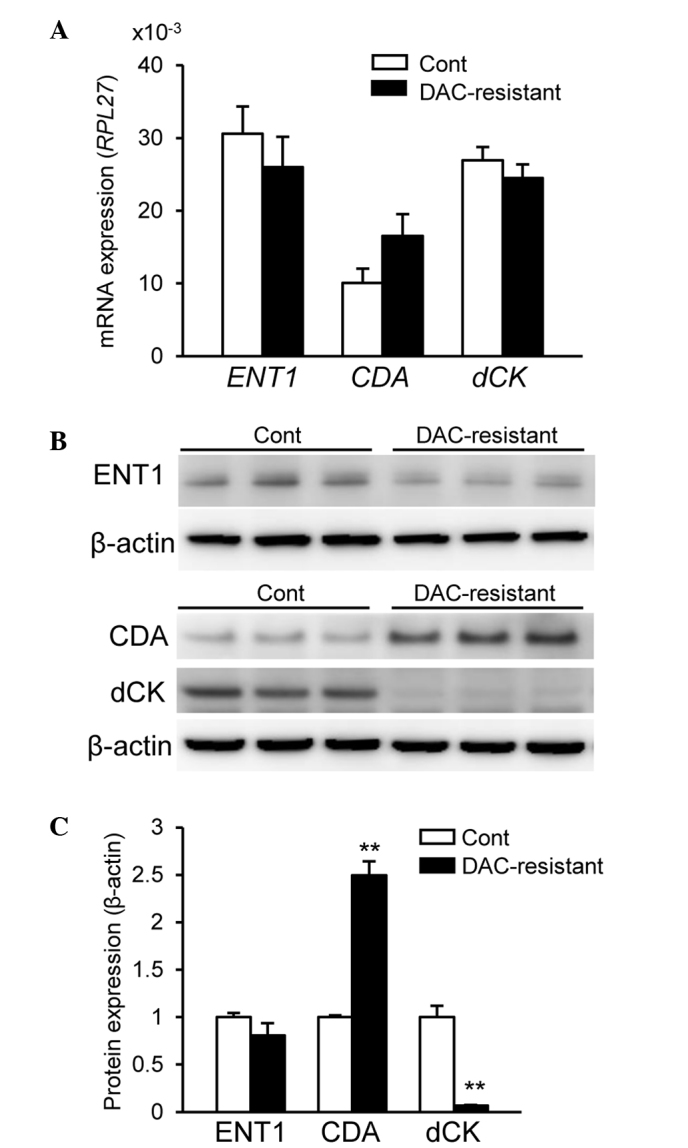
Altered CDA and dCK protein expression levels in DAC-resistant HCT116 cells. (A) mRNA expression levels of *ENT1*, *CDA* and *dCK* in control and DAC-resistant HCT116 cells were analyzed by reverse transcription quantitative polymerase chain reaction and normalized to *RPL27* mRNA expression. (B) Protein expression levels of ENT1, CDA and dCK in control and DAC-resistant HCT116 cells were analyzed by western blotting. β-actin was used as a loading control. (C) Quantification of protein expression by Image J. Values are presented as the mean ± standard error of the mean of three independent experiments. **P<0.01 vs. control HCT116 cells. DAC, decitabine; ENT1, equilibrative nucleoside transporter 1; CDA, cytidine deaminase; dCK, deoxycytidine kinase; *RPL27, ribosomal protein 27*; cont, control.

In order to elucidate the mechanisms underlying acquired resistance to DAC, the mRNA expression levels of *ENT1*, *CDA* and *dCK were examined*. As shown in Fig. 2A, no significant differences were observed in the expression levels of these mRNAs between control and DAC-resistant HCT116 cells (P>0.05). However, the expression of *ENT2*, *ENT3* and *CNTs* mRNA was markedly reduced compared with that of *ENT1* mRNA in HCT116 cells (data not shown). The protein levels of ENT1, CDA and dCK were subsequently determined. No significant differences were noted in ENT1 protein expression levels between control and DAC-resistant HCT116 cells (Fig. 2B and C). However, CDA protein expression levels were significantly increased and dCK protein expression levels were significantly attenuated in DAC-resistant HCT116 cells compared with control HCT116 cells (P<0.01) (Fig. 2B and C). By contrast, CDA protein expression levels were markedly reduced in HT29 cells compared with HCT116 cells, whereas ENT1 and dCK proteins were expressed in HT29 cells (data not shown). These results indicate the involvement of CDA and dCK in the acquired resistance to DAC by HCT116 cells.

## Discussion

Numerous clinical trials have demonstrated the therapeutic potential of DAC in hematological malignancies (29–31); however, according to clinical trials using DAC, fewer responses were reported in solid tumors for DAC compared with AC, indicating a reduced efficacy (17). One difficulty associated with treating solid tumors over hematological malignancies is due to the lack of dividing cells in solid tumors, as DAC and AC are S-phase-specific drugs and DNA incorporation is essential in order to exert their epigenetic effects (17). However, previous studies have reported the stable and altered expression of genes in solid tumors, which supported the activity of DAC and AC due to the abundance of epigenetic aberrations observed in solid tumors (17,32). Although the efficacy of DAC for solid tumors, including CRC, remains controversial, combination therapy with DAC and cytotoxic anticancer drugs may be beneficial (18,19). It was previously reported that DAC exhibited a synergic effect on the cytotoxicity induced by anticancer drugs in human CRC cells (20). Thus, research using CRC cells with acquired resistance to DAC may be important for elucidating the mechanisms underlying resistance to DAC as well as cross-resistance to other drugs.

The concentration of DAC required to inhibit DNA methylation in clinical applications was reported to be ~0.3 µM, which is the maximal plasma concentration in humans (33). This concentration was unchanged during repeated dosing due to the short terminal phase elimination t_1/2_ value, ~35 min (33). It was previously reported that human HCT116 CRC cells exhibited the highest sensitivity to DAC among four human CRC cell lines examined (20). In the present study, increasing concentrations of DAC (10–540 nM) were used to induce DAC resistance in HCT116 cells. The results demonstrated an increased IC_50_ value of DAC following treatment for 81 days. This result corresponded with the alterations in *SFRP1* mRNA and DNMT1 protein expression levels. Thus, long-term treatment with DAC, at concentrations that inhibited DNMT1, resulted in acquired DAC resistance in HCT116 cells.

CRC cells that acquired resistance to HDAC inhibitors, including TSA, SAHA and VPA, were also reported to exhibit cross-resistance to different HDAC inhibitors (7–9). However, in the present study, DAC-resistant HCT116 cells demonstrated no cross-resistance to DNMT inhibitors, AC and Zeb. These results suggested that the long-term treatment with DAC did not affect UCK, a key enzyme for the activation of AC and Zeb (12,34). Furthermore, DAC-resistant HCT116 cells did not exhibit cross-resistance to any of the HDAC inhibitors, including TSA, SAHA and VPA. This result implied that the long-term treatment with DAC did not influence the pathways involved in histone modification. In addition, long-term treatment with DAC did not alter the sensitivity of DAC-resistant HCT116 cells to anticancer drugs used to treat CRC, including 5-FU, CPT-11, SN-38 and L-OHP. These results indicated that long-term DAC treatment may not affect the pathways involved in the effects of anticancer drugs, including apoptosis and the cell cycle as well as the transport and metabolism of these drugs (35,36). By contrast, the present study demonstrated that the sensitivity of Gem was markedly lower in DAC-resistant HCT116 cells compared with control HCT116 cells. The intracellular uptake of Gem is primarily mediated by ENT1 (28). Gem is phosphorylated to its monophosphate form by dCK in cells and this step is essential for further phosphorylation to its active triphosphate form (28). Gem is known to be primarily inactivated by the transformation of CDA into 2′,2′-difluorodeoxyuridine (28). The mechanisms underlying Gem resistance have been extensively examined and are known to involve ENT1, CDA and dCK (37,38). The mechanisms responsible for DAC resistance may be similar to those for Gem resistance, as DAC-resistant HCT116 cells were more resistant to Gem compared with control HCT116 cells in the current study. In addition, increased CDA and decreased dCK protein expression levels were observed in the present study, whereas the protein expression of ENT1 was not markedly changed. Only ENT1 protein expression levels were examined in the present study, as preliminary studies revealed that the expression of *ENT1* mRNA was markedly higher than that of *ENT2*, *ENT3* and *CNTs* mRNA in HCT116 cells (data not shown). However, the present study demonstrated that no significant differences were observed in the mRNA expression of *ENT1, CDA* and *dCK* between DAC-resistant HCT116 cells and control HCT116 cells. A previous study demonstrated that DAC was able to interact with all nucleoside transporter proteins with high affinity; however, it was reported that DAC is not more efficiently transported via nucleoside transporter-type proteins compared with other nucleosides and analogs (uridine, adenosine, Gem and AC) (14,16). These results therefore suggested that the involvement of ENT1 in the acquisition of resistance to DAC in HCT116 cells may be negligible. Another previous study reported that the resistance of HL60 cells to DAC induced by DAC exposure was associated with attenuated dCK levels due to dCK mutations (11). Regarding Gem, reduced phosphorylation by dCK was identified to be a key process for acquiring Gem resistance over other processes via ENT1 and CDA in a pancreatic cancer cell line (39). In addition, the deaminated metabolite of Gem by CDA was suggested to modulate the rate of Gem transport and intracellular phosphorylation via dCK (40). Thus, an increase in the expression of CDA may partly affect the sensitivity to Gem or DAC. Alterations in CDA and dCK may therefore contribute to DAC resistance and cross-resistance to Gem. However, further studies are required in order to clarify the detailed mechanisms underlying acquired resistance to DAC, such as differences in the contribution of CDA and dCK, microRNA expression and gene mutations. It was previously reported that the IC_50_ value of DAC in HT29 cells, another CRC cell line, was ~1,400 µM, which was markedly higher compared with DAC-resistant HCT116 cells (20). The results of preliminary studies demonstrated that CDA protein expression levels were markedly lower in HT29 cells compared with HCT116 cells, whereas ENT1 and dCK proteins were expressed in HT29 cells (data not shown). Therefore, mechanisms that do not involve ENT1, CDA and dCK may have contributed to the low sensitivity of HT29 cells to DAC. Further studies are required in order to elucidate the differences in the mechanisms underlying resistance to DAC among cell lines.

In conclusion, the results of the present study demonstrated that the long-term treatment of HCT116 cells with DAC increased the expression of CDA and decreased that of dCK, which indicated the potential role of these proteins in the acquisition of resistance to DAC. Furthermore, DAC-resistant HCT116 cells exhibited cross-resistance to Gem; however, cross-resistance was not observed with other DNMT inhibitors (AC and Zeb), HDAC inhibitors (TSA, SAHA and VPA) or anticancer drugs (5-FU, CPT-11 and L-OHP).

## Figures and Tables

**Table I.** IC_50_ values of DAC in HCT116 cells following long-term treatment with DAC.

DAC treatment	IC_50_ (µM)	Relative resistance
Control	8.6±2.7	1.0
Day 28	4.3±1.7	0.5
Day 52	12.2±3.8	1.4
Day 81	872.4±152.0	101.4
Day 104	884.2±85.9	102.8

Values are presented as the mean ± standard error of the mean of three independent experiments. Relative resistance = IC_50_(DAC-treated cells)/IC_50_(control cells). DAC, decitabine.

**Table II.** IC_50_ values of epigenetic modifiers in control and DAC-resistant HCT116 cells.

Epigenetic modifiers	AC (µM)	Zeb (µM)	TSA (nM)	SAHA (µM)	VPA (mM)
Control	18.28±0.56	1040.15±73.21	23.99±4.92	0.64±0.05	1.22±0.02
DAC-resistant	16.36±0.88	918.11±85.72	17.15±1.70	0.48±0.04	0.94±0.04
Relative resistance	0.89	0.88	0.71	0.75	0.77
P-value	0.14	0.34	0.26	0.05	2.30×10^−3a^

Values are presented as the mean ± standard error of the mean of three independent experiments. Resistance = IC_50_(DAC-treated cells)/IC_50_(control cells).

a P<0.05 indicates statistical significance. DAC, decitabine; AC, azacitidine; Zeb, zebularine; TSA, trichostatin A; SAHA, vorinostat; VPA, valproic acid.

**Table III.** IC_50_ values of anticancer drugs in control and DAC-resistant HCT116 cells.

Anticancer drugs	5-FU (µM)	CPT-11 (µM)	SN-38 (nM)	L-OHP (µM)	Gem (nM)
Control	3.34±0.21	2.40±0.20	2.82±0.14	0.71±0.12	7.77±0.22
DAC-resistant	2.79±0.15	1.73±0.12	1.91±0.28	0.64±0.06	249.44±8.59
Relative resistance	0.84	0.72	0.68	0.90	32.10
P-value	0.10	0.05^a^	0.04^a^	0.63	9.5×10^−6 a^

Values are presented as the mean ± standard error of the mean of three independent experiments. Resistance = IC_50_(DAC-treated cells)/IC_50_(control cells).

a P<0.05 indicates statistical significance. DAC, decitabine; 5-FU, 5-fluorouracil; CPT-11, irinotecan; SN-38, 7-ethyl-10-hydroxycamptothecin; L-OHP, oxaliplatin; Gem, gemcitabine.

## References

[1] Sarkar S, Horn G, Moulton K (2013). Cancer development, progression, and therapy: An epigenetic overview. Int J Mol Sci.

[2] Ishikawa T (2014). Novel therapeutic strategies using hypomethylating agents in the treatment of myelodysplastic syndrome. Int J Clin Oncol.

[3] Foulks JM, Parnell KM, Nix RN (2012). Epigenetic drug discovery: Targeting DNA methyltransferases. J Biomol Screen.

[4] Christman JK (2002). 5-Azacytidine and 5-aza-2′-deoxycytidine as inhibitors of DNA methylation: Mechanistic studies and their implications for cancer therapy. Oncogene.

[5] Pluchino KM, Hall MD, Goldsborough AS (2012). Collateral sensitivity as a strategy against cancer multidrug resistance. Drug Resist Updat.

[6] Stordal B, Pavlakis N, Davey R (2007). Oxaliplatin for the treatment of cisplatin-resistant cancer: A systematic review. Cancer Treat Rev.

[7] Fedier A, Dedes KJ, Imesch P (2007). The histone deacetylase inhibitors suberoylanilide hydroxamic (Vorinostat) and valproic acid induce irreversible and MDR1-independent resistance in human colon cancer cells. Int J Oncol.

[8] Dedes KJ, Dedes I, Imesch P (2009). Acquired vorinostat resistance shows partial cross-resistance to ‘second-generation’ HDAC inhibitors and correlates with loss of histone acetylation and apoptosis but not with altered HDAC and HAT activities. Anticancer Drugs.

[9] Imesch P, Dedes KJ, Furlato M, Fink D, Fedier A (2009). MLH1 protects from resistance acquisition by the histone deacetylase inhibitor trichostatin A in colon tumor cells. Int J Oncol.

[10] Qin T, Castoro R, El Ahdab S, Jelinek J, Wang X, Si J, Shu J, He R, Zhang N, Chung W, Kantarjian HM, Issa JP (2011). Mechanisms of resistance to decitabine in the myelodysplastic syndrome. PLoS One.

[11] Qin T, Jelinek J, Si J, Shu J, Issa JP (2009). Mechanisms of resistance to 5-aza-2′-deoxycytidine in human cancer cell lines. Blood.

[12] Sripayap P, Nagai T, Uesawa M, Kobayashi H, Tsukahara T, Ohmine K, Muroi K, Ozawa K (2014). Mechanisms of resistance to azacitidine in human leukemia cell lines. Exp Hematol.

[13] Mahfouz RZ, Jankowska A, Ebrahem Q, Gu X, Visconte V, Tabarroki A, Terse P, Covey J, Chan K, Ling Y, Engelke KJ (2013). Increased CDA expression/activity in males contributes to decreased cytidine analog half-life and likely contributes to worse outcomes with 5-azacytidine or decitabine therapy. Clin Cancer Res.

[14] Damaraju VL, Mowles D, Yao S, Ng A, Young JD, Cass CE, Tong Z (2012). Role of human nucleoside transporters in the uptake and cytotoxicity of azacitidine and decitabine. Nucleosides Nucleotides Nucleic Acids.

[15] Hummel-Eisenbeiss J, Hascher A, Hals PA, Sandvold ML, Müller-Tidow C, Lyko F, Rius M (2013). The role of human equilibrative nucleoside transporter 1 on the cellular transport of the DNA methyltransferase inhibitors 5-azacytidine and CP-4200 in human leukemia cells. Mol Pharmacol.

[16] Arimany-Nardi C, Errasti-Murugarren E, Minuesa G, Martinez-Picado J, Gorboulev V, Koepsell H, Pastor-Anglada M (2014). Nucleoside transporters and human organic cation transporter 1 determine the cellular handling of DNA-methyltransferase inhibitors. Br J Pharmacol.

[17] Cowan LA, Talwar S, Yang AS (2010). Will DNA methylation inhibitors work in solid tumors? A review of the clinical experience with azacitidine and decitabine in solid tumors. Epigenomics.

[18] Azad N, Zahnow CA, Rudin CM, Baylin SB (2013). The future of epigenetic therapy in solid tumours - lessons from the past. Nat Rev Clin Oncol.

[19] Flis S, Gnyszka A, Flis K (2014). DNA methyltransferase inhibitors improve the effect of chemotherapeutic agents in SW48 and HT-29 colorectal cancer cells. PLoS One.

[20] Ikehata M, Ogawa M, Yamada Y, Tanaka S, Ueda K, Iwakawa S (2014). Different effects of epigenetic modifiers on the cytotoxicity induced by 5-fluorouracil, irinotecan or oxaliplatin in colon cancer cells. Biol Pharm Bull.

[21] Baylin SB, Ohm JE (2006). Epigenetic gene silencing in cancer - a mechanism for early oncogenic pathway addiction?. Nat Rev Cancer.

[22] Ahmed D, Eide PW, Eilertsen IA, Danielsen SA, Eknæs M, Hektoen M, Lind GE, Lothe RA (2013). Epigenetic and genetic features of 24 colon cancer cell lines. Oncogenesis.

[23] Issa JP (2004). CpG island methylator phenotype in cancer. Nat Rev Cancer.

[24] Kakumoto M, Takara K, Sakaeda T, Tanigawara Y, Kita T, Okumura K (2002). MDR1-mediated interaction of digoxin with antiarrhythmic or antianginal drugs. Biol Pharm Bull.

[25] de Jonge HJ, Fehrmann RS, de Bont ES, Hofstra RM, Gerbens F, Kamps WA, de Vries EG, van der Zee AG, te Meerman GJ, ter Elst A (2007). Evidence based selection of housekeeping genes. PLoS One.

[26] Wong ML, Medrano JF (2005). Real-time PCR for mRNA quantitation. Biotechniques.

[27] Suzuki H, Watkins DN, Jair KW, Schuebel KE, Markowitz SD, Chen WD, Pretlow TP, Yang B, Akiyama Y, Van Engeland M, Toyota M (2004). Epigenetic inactivation of SFRP genes allows constitutive WNT signaling in colorectal cancer. Nat Genet.

[28] Ueno H, Kiyosawa K, Kaniwa N (2007). Pharmacogenomics of gemcitabine: Can genetic studies lead to tailor-made therapy?. Br J Cancer.

[29] Lyons J, Bayar E, Fine G, McCullar M, Rolens R, Rubinfeld J, Rosenfeld C (2003). Decitabine: Development of a DNA methyltransferase inhibitor for hematological malignancies. Curr Opin Investig Drugs.

[30] Kihslinger JE, Godley LA (2007). The use of hypomethylating agents in the treatment of hematologic malignancies. Leuk Lymphoma.

[31] Robak T (2011). New nucleoside analogs for patients with hematological malignancies. Expert Opin Investig Drugs.

[32] Qiu X, Hother C, Ralfkiær UM, Søgaard A, Lu Q, Workman CT, Liang G, Jones PA, Grønbæk K (2010). Equitoxic doses of 5-azacytidine and 5-aza-2′deoxycytidine induce diverse immediate and overlapping heritable changes in the transcriptome. PLoS One.

[33] Cashen AF, Shah AK, Todt L, Fisher N, DiPersio J (2008). Pharmacokinetics of decitabine administered as a 3-h infusion to patients with acute myeloid leukemia (AML) or myelodysplastic syndrome (MDS). Cancer Chemother Pharmacol.

[34] Marquez VE, Kelley JA, Agbaria R, Ben-Kasus T, Cheng JC, Yoo CB, Jones PA (2005). Zebularine: A unique molecule for an epigenetically based strategy in cancer chemotherapy. Ann NY Acad Sci.

[35] Panczyk M (2014). Pharmacogenetics research on chemotherapy resistance in colorectal cancer over the last 20 years. World J Gastroenterol.

[36] Damia G, Broggini M (2004). Cell cycle checkpoint proteins and cellular response to treatment by anticancer agents. Cell Cycle.

[37] Mini E, Nobili S, Caciagli B, Landini I, Mazzei T (2006). Cellular pharmacology of gemcitabine. Ann Oncol.

[38] Kahramanoğullari O, Fantaccini G, Lecca P, Morpurgo D, Priami C (2012). Algorithmic modeling quantifies the complementary contribution of metabolic inhibitions to gemcitabine efficacy. PLoS One.

[39] Ohmine K, Kawaguchi K, Ohtsuki S, Motoi F, Egawa S, Unno M, Terasaki T (2012). Attenuation of phosphorylation by deoxycytidine kinase is key to acquired gemcitabine resistance in a pancreatic cancer cell line: Targeted proteomic and metabolomic analyses in PK9 cells. Pharm Res.

[40] Hodge LS, Taub ME, Tracy TS (2011). The deaminated metabolite of gemcitabine, 2′,2′-difluorodeoxyuridine, modulates the rate of gemcitabine transport and intracellular phosphorylation via deoxycytidine kinase. Drug Metab Dispos.

